# An unusual case of intestinal obstruction due to abdominal cocoon: A case report

**DOI:** 10.1016/j.ijscr.2021.106282

**Published:** 2021-08-04

**Authors:** Om Prakash Bhatta, Rupesh Verma, Gyaneswor Shrestha, Deepak Sharma, Romi Dahal, Prasan Bir Singh Kansakar

**Affiliations:** a Maharajgunj Medical Campus, Institute of Medicine, Kathmandu, Nepal; b Department of GI and General Surgery, Tribhuvan University Teaching Hospital, Institute of Medicine, Kathmandu, Nepal

**Keywords:** Abdominal cocoon, Case report, Encapsulating Peritoneal Sclerosis, Intestinal obstruction, Peritoneal fibrosis

## Abstract

**Introduction and importance:**

Abdominal cocoon (AC) or Encapsulating Peritoneal Sclerosis (EPS) is a rare cause of bowel obstruction and due to non-specific presentation, it can be misdiagnosed and often mistreated.

**Case presentation:**

We present the case of 42 years male with a history suggestive of complete small bowel obstruction (SBO) without a history of pulmonary tuberculosis (TB) or peritoneal dialysis. CT imaging as well as the intraoperative finding of a cocoon membrane encasing the small bowel led to the diagnosis of abdominal cocoon.

**Clinical discussion:**

Abdominal cocoon can be idiopathic or secondary to peritoneal dialysis, tuberculosis, or other rare causes. Patients usually present with features of SBO with varying severity. Diagnosis is aided by imaging investigations mainly CT scan and management is primarily surgical and usually involves adhesiolysis, total removal of the membrane with or without bowel loop resection.

**Conclusion:**

Diagnosis of abdominal cocoon warrants awareness of the disease and a high index of suspicion of the treating clinician in patients with intestinal obstruction and an abdominal lump without a history of previous abdominal surgery. CT can guide diagnosis and early operative management seems to bear the best outcomes.

## Highlights

• Abdominal cocoon is a rare cause of intestinal obstruction with high morbidity and mortality if not diagnosed in time.
• We report a case of an abdominal cocoon in an adult male without any obvious preceding cause.
• Diagnosis warrants a high index of suspicion in patients with features of intestinal obstruction and an abdominal lump.
• CT can guide diagnosis and early operative management seems to bear the best outcomes.

## 1 Introduction

Small-bowel obstruction (SBO) is one of the commonest surgical emergencies presenting to the emergency department [1]. Adhesions or bands are the leading cause of bowel obstruction followed by obstructed or strangulated hernias, tumors leading to mass effects or stricture, intestinal tuberculosis, volvulus, and intussusception [2].

Abdominal cocoon (AC) or Encapsulating Peritoneal Sclerosis (EPS) is a rare cause of bowel obstruction and includes the formation of a fibro collagenous membrane encasing the small intestine in the manner of a cocoon. EPS may be idiopathic or associated with peritoneal dialysis, TB infection of the abdomen, and other rare causes.

We present a case of complete small bowel obstruction due to cocoon formation who underwent operative management. This case has been reported in line with the SCARE checklist [3].

## 2 Case

We present the case of 42-year-old male with a history of right upper quadrant abdominal pain, vomiting, and inability to pass stool and flatus for two days. The pain was acute in onset, severe, and associated with four episodes of non-bilious vomiting. With these symptoms, he noticed a non-tender, firm lump palpable in the right upper quadrant. There was no past history of pulmonary TB or peritoneal dialysis.

He had a similar episode one month back which was managed conservatively.

Per abdominal examination revealed a soft non-distended abdomen with a visible intraperitoneal mass about 4 × 5 cm over the right lumbar region. Bowel sounds were exaggerated.

Laboratory investigations were sent which showed leukocytosis (total leukocyte count 15.7 × 10^9^/l, neutrophils 65%, lymphocytes 30%) with rest parameters being normal. Plain radiographs of the abdomen showed multiple air-fluid levels with dilated small bowel loops. Chest radiograph was unremarkable and sputum Acid Fast Bacilli stain reported negative. TB was not detected in Gene-Xpert of sputum. Ultrasonography of the abdomen and pelvis showed dilated bowel loops but characteristic trilaminar membrane was not noted.

**Fig. 1:**
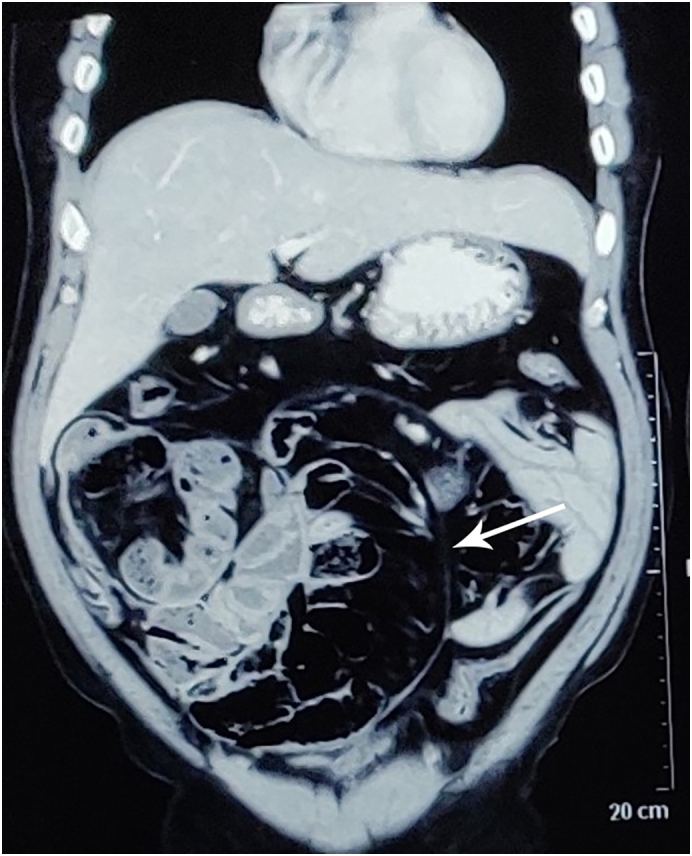
CT image showing the cocoon membrane encasing the bowel loops (arrow).

CT abdomen and pelvis, with and without contrast, showed (see Fig. 1) small bowel loops clustered and dilated measuring 33 mm in diameter and seen on the right side of the abdomen encapsulated in a thin membrane with mild adjacent free fluid. Mesenteric vessels were noted to be stretched around these dilated bowel loops.

**Fig. 2:**
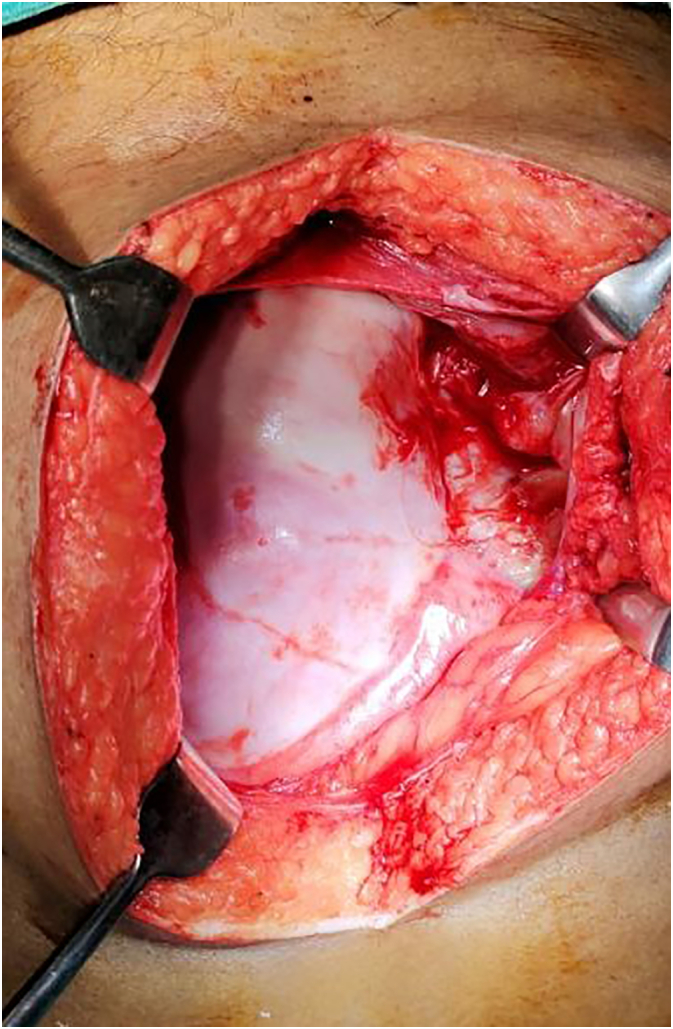
Intraoperative image showing the cocoon membrane encasing the bowel.

The patient underwent exploratory laparotomy and intraoperatively (see Fig. 2) there was an opaque membrane encasing the whole of the small bowel suggestive of the abdominal cocoon with dense interloop adhesion along with 10 cm gangrenous ileal loop about 5 cm proximal to ileocecal junction. With these findings, the patient underwent right limited hemicolectomy with adhesiolysis and end-to-end ileocolic anastomosis.

The postoperative hospital course was uneventful, and the patient was discharged on the 7th postoperative day.

**Fig. 3:**
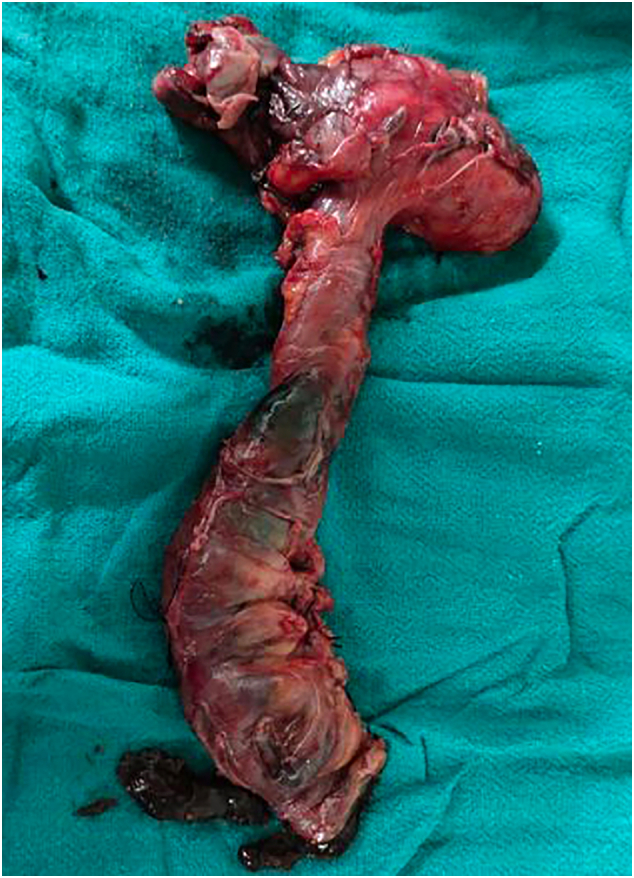
Gross specimen showing gangrenous terminal ileum.

The resected specimen (see Fig. 3) showed gangrenous terminal ileum with no other gross pathology noted.

The histopathological report showed fibro collagenous tissue with moderate chronic inflammatory infiltrate. There were no granulomas or malignant cells. The patient was asymptomatic after 2 months follow-up period.

## 3 Discussion

Encapsulating Peritoneal Sclerosis is a rare cause of intestinal obstruction. It was first described by Foo et al. in 1978 as an abdominal cocoon in a case series of 10 cases of young postmenarchal females and postulated it to be the result of retrograde menstruation [4].

The exact etiology of EPS is lacking, but it can be classified into primary or idiopathic form and secondary form. The idiopathic form is less common and hypothesized to result from subclinical primary viral peritonitis, as an immunologic reaction to gynecologic infections, or because of retrograde menstruation [5].

The secondary form of EPS is related to conditions causing chronic peritoneal irritation and inflammation with consequent fibrosis and the formation of a cocoon membrane. The cause of such inflammation has been hypothesized to be peritoneal dialysis, tuberculosis, local irritation due to trauma or surgery, intraperitoneal therapy, ventriculoperitoneal shunt, and infectious peritonitis. Other rare causes inflicted to cause EPS are liver cirrhosis, sarcoidosis, gastrointestinal tumors, SLE, fibrogenic foreign body and orthotopic liver transplantation [5], [6], [7], [8].

Patients in TB endemic regions must be accordingly evaluated for TB with appropriate tests [5].

Although exact pathogenesis is not well understood, it is believed to be a result of peritoneal irritation leading to inflammation and a consequent proliferation and hyperplasia of peritoneal mesothelial cells and peritoneal capillary angiogenesis. The increased fibrogenesis and endothelial permeability lead to marked fibrin deposition in the peritoneum forming a dense capsule encasing whole or part of bowel giving a characteristic appearance to the disease. The inflammatory trigger could be any of the above-mentioned etiological agents [12], [19].

In this case, features suggestive of EPS along with the intraoperative finding of a cocoon membrane encasing the whole of small bowel without any secondary cause led to the diagnosis of type II primary abdominal cocoon.

Patients usually present with episodes of small bowel obstruction which may be acute, subacute, or chronic, and symptoms are related to duration and severity of obstruction. Cardinal features might not be present in all cases [5], [9], [10], [11].

Several imaging modalities can be used to aid in the diagnosis of EPS but only laparotomy and histopathological findings seem to be confirmatory [9].

Plain abdominal x-rays may show features of small bowel obstruction and calcifications in case of peritoneal dialysis [12].

USG may show the thick hypoechoic membrane surrounding the dilated loops of the bowel with disturbed motility, tethering of the bowel to the posterior abdominal wall, trilaminar membrane appearance, and intraperitoneal echogenic strands [13], [14], [15], [16].

CT scan of the abdomen is highly sensitive for the diagnosis and shows small bowel loops congregated and partially or fully encased by a thick fibrous membrane-like sac. Besides this CT might show signs of disturbed motility, thickening of the intestinal wall, calcifications, peritoneal thickening, and enhancement. CT scan is also useful to rule out complications of EPS as well as other causes of intestinal obstruction, and the advent of multi-detector CT with an improved quality high-resolution image and excellent multiplanar reconstructions has led to higher preoperative diagnoses [5], [17], [18], [19].

Intraoperative findings include opaque and thickened peritoneum with cocoon membrane partially or completely encasing the bowel loops, with other findings dependent on the underlying etiology [19], [20].

Biopsy shows thickened fibro collagenous tissue with or without chronic inflammation in the form of lymphocytes and plasma cell infiltrates [10].

Causes of SBO with a similar radiologic appearance include internal hernia, pseudomyxoma peritonei, congenital peritoneal encapsulation, peritoneal mesothelioma, tuberculous peritonitis, peritoneal carcinomatosis, sclerosing malignant lymphoma, and malignant primary mesenteric tumors [13], [19], [20].

Management of EPS depends on the underlying etiology if evident and in the severity of symptoms. In the case of asymptomatic patients or those with mild symptoms, it can be treated conservatively similar to that of SBO. Studies in EPS due to peritoneal dialysis show variable benefits of corticosteroids, tamoxifen, and immunosuppressive agents along with supportive treatment [7]. However, management of EPS in symptomatic cases with advanced stage intestinal obstruction is primarily surgical with choice of surgery based on the extent of bowel involvement and involves adhesiolysis, total removal of the membrane with or without bowel loop resection [7], [9].

Postoperative complications include early postoperative small bowel obstruction and rarely intra-abdominal infection, intestinal fistula, short bowel syndrome, or bowel perforation. Overall good prognosis is reported with rare chances of recurrence [10].

In a series of 24 cases, patients usually presented with partial or complete intestinal obstruction and an abdominal mass, 4 cases were diagnosed preoperatively by imaging studies, the rest 20 were diagnosed during laparotomy. All the patients underwent operative management, following which most patients had uneventful postoperative recovery and there were no recurrences in a mean follow-up period of 37 months [10].

## 4 Conclusions

Abdominal cocoon syndrome is a rare cause of intestinal obstruction, but can have high morbidity and mortality if not diagnosed in time and managed properly. Diagnosis warrants awareness of the disease and a high index of suspicion of the treating clinician in patients with intestinal obstruction and an abdominal lump. CT can guide diagnosis and early operative management seems to bear the best outcomes.

## Provenance and peer review

Not commissioned, externally peer-reviewed.

## Consent

Written informed consent was obtained from the patient for publication of this case report and accompanying images. A copy of the written consent is available for review by the Editor-in-Chief of this journal on request.

## Ethical approval

Not required.

## Funding

None.

## Guarantor

Om Prakash Bhatta.

## Research registration number

Not applicable.

## CRediT authorship contribution statement

Prasan Bir Singh Kansakar (PBK), Romi Dahal (RD), Deepak Sharma (DS), Rupesh Verma (RV), Gyaneswor Shrestha (GS) = Study concept, Data collection, and Surgical therapy for the patient
Om Prakash Bhatta (OPB), RV, GS = Writing - original draft preparation and editing
PBK, RD, DS = Senior author and Manuscript reviewer.

All authors critically reviewed, revised and contributed to the final article. All authors read and approved the final manuscript.

## Declaration of competing interest

Nothing to declare.
